# Impact of Hepatoma-Derived Growth Factor Blockade on Resiniferatoxin-Induced Neuropathy

**DOI:** 10.1155/2021/8854461

**Published:** 2021-02-27

**Authors:** Chieh-Hsin Wu, Ming-Kung Wu, Chun-Ching Lu, Hung-Pei Tsai, Ying-Yi Lu, Chih-Lung Lin

**Affiliations:** ^1^Division of Neurosurgery, Department of Surgery, Kaohsiung Medical University Hospital, Kaohsiung 807, Taiwan; ^2^Department of Surgery, School of Medicine, College of Medicine, Kaohsiung Medical University, Kaohsiung 807, Taiwan; ^3^Department of Psychiatry, Kaohsiung Chang Gung Memorial Hospital and Chang Gung University College of Medicine, Kaohsiung 833, Taiwan; ^4^Department of Orthopaedics and Traumatology, Taipei Veterans General Hospital, Taipei 112, Taiwan; ^5^Faculty of Medicine, National Yang Ming Chiao Tung University, Taipei 112, Taiwan; ^6^Department of Dermatology, Kaohsiung Veterans General Hospital, Kaohsiung 813, Taiwan; ^7^Graduate Institute of Medicine, College of Medicine, Kaohsiung Medical University, Kaohsiung 807, Taiwan; ^8^Shu-Zen Junior College of Medicine and Management, Kaohsiung 821, Taiwan

## Abstract

Resiniferatoxin is an ultrapotent capsaicin analog that mediates nociceptive processing; treatment with resiniferatoxin can cause an inflammatory response and, ultimately, neuropathic pain. Hepatoma-derived growth factor, a growth factor related to normal development, is associated with neurotransmitters surrounding neurons and glial cells. Therefore, the study aims to investigate how blocking hepatoma-derived growth factor affects the inflammatory response in neuropathic pain. Serum hepatoma-derived growth factor protein expression was measured via ELISA. Resiniferatoxin was administrated intraperitoneally to induce neuropathic pain in 36 male Sprague-Dawley rats which were divided into three groups (resiniferatoxin+recombinant hepatoma-derived growth factor antibody group, resiniferatoxin group, and control group) (*n* = 12/group). The mechanical threshold response was tested with calibration forceps. Cell apoptosis was measured by TUNEL assay. Immunofluorescence staining was performed to detect apoptosis of neuron cells and proliferation of astrocytes in the spinal cord dorsal horn. RT-PCR technique and western blot were used to measure detect inflammatory factors and protein expressions. Serum hepatoma-derived growth factor protein expression was higher in the patients with sciatica compared to controls. In resiniferatoxin-group rats, protein expression of hepatoma-derived growth factor was higher than controls. Blocking hepatoma-derived growth factor improved the mechanical threshold response in rats. In dorsal root ganglion, blocking hepatoma-derived growth factor inhibited inflammatory cytokines. In the spinal cord dorsal horn, blocking hepatoma-derived growth factor inhibited proliferation of astrocyte, apoptosis of neuron cells, and attenuated expressions of pain-associated proteins. The experiment showed that blocking hepatoma-derived growth factor can prevent neuropathic pain and may be a useful alternative to conventional analgesics.

## 1. Introduction

After peripheral nerve degeneration, nociceptive input was reduced and neuropathic pain was developed. Sciatica, which is characterized by radicular leg pain radiating along the sciatic distribution, is an exemplary cause of neuropathic pain [1, 2]. Sensory nerve fibers in the peripheral nerves are axonal processes of dorsal root ganglion (DRG) neurons composed of large- and small-size neurons with corresponding diameters of nerve fibers. By affecting nociceptive nerves with small-diameter, some patients with peripheral nerve diseases have paradoxical symptoms such as neuropathic pain combined with skin denervation that reduces sensitivity to noxious stimuli [3, 4]. Similar phenomena may result from capsaicin-induced skin denervation [5, 6]. However, it is unclear whether injury to only small neurons can cause this paradoxical combination of reduced neuropathic pain and nociception. Therefore, this study used a rat model of neuropathic pain to investigate whether treatment with the ultrapotent capsaicin analog resiniferatoxin (RTX) induces paradoxical changes in mechanical sensitivities [7, 8]. Unmyelinated nerves degeneration together with denervation of skin is the main feature of RTX-induced neuropathy [9], which is a precursor of pure small-fiber neuropathy and a good model of neuropathic pain. Studies show RTX can induce neuropathic pain through interaction with and upregulation of tumor necrosis factor *α* (TNF-*α*) [10], which then induces cell apoptosis through an inflammatory response [11, 12]. After nerve injury and inflammation, activated glial cells in the central nervous system express TNF-*α*, which then induces the release of proapoptotic factors [11, 12]. In a previous study, a global analysis that integrated proteome analysis revealed that a lack of hepatoma-derived growth factor (HDGF) genes can limit apoptosis induced by TNF-*α* [11].

The HDGF is a 240 amino-acid protein isolated from human hepatoma cells [13]. Surface expressed nucleoli have recently been identified as HDGF receptors [14]. During cell development, HDGF stimulates cell proliferation in fibroblasts, endothelial cells, and hepatoma cells [15]. The HDGF contributes to the normal development and regeneration of the liver [16–18] and to the development of the lungs [19, 20], vascular system [21–23], and heart [24]. This multifunctional protein also has important roles in cancer development due to its participation in various cellular events such as processing of RNA, ribosome biogenesis, transcriptional regulation, and damage repair of DNA [25]. Many studies have also documented the role of HDGF as a mitogen with extracellular proliferative effects on hepatoma cells, fibroblasts, vascular smooth muscle cells, and endothelial cells [19]. Additionally, HDGF is reportedly synthesized and localized mainly in brain neurons, which is retained in nucleus and released under necrotic status. It also upregulated in astrocytes exposed to neural cell adhesion molecules and has mitogenic and/or inflammatory effects on glial cells. Since glial cells express HDGF when activated and/or stimulated by certain signals, HDGF may function as a mitogenic factor in glial cells or an autocrine neurotrophic factor in neighboring neurons [26].

We hypothesized that transduction of pain signals to activated glial cells in the spinal cord produces an abundance of TNF-*α*, which then causes RTX-induced neuropathic pain. A rat model was used to investigate the role of recombinant HDGF antibody (rHDGF antibody) in attenuating pain behaviors.

## 2. Materials and Methods

### 2.1. Patient Serum Collection

Patients with sciatica secondary to disc herniation were recruited from our hospital. The inclusion criteria were age more than 20 years, lumbar disc herniation verified by magnetic resonance imaging, and a dermatomal distribution of pain in a lower limb. Exclusion criteria were any history of spinal infections, spinal tumor, or other refractory neuropathic pain. The inclusion criteria for the control group were age more than 20 years and no sciatic pain. All participants signed informed consent forms. Serum was collected, centrifuged at 3,000 × rpm for 10 minutes at 4°C, and stored at -80°C until analysis. The study design was evaluated and approved by the Institutional Review of Board of Kaohsiung Medical University Hospital (KMUHIRB-20140262).

### 2.2. Preparation of rHDGF Antibody

The rHDGF antibody was customized by Leadgene Biomedical Inc. (Tainan, Taiwan). 50 *μ*g recombinant rat HDGF (rrHDGF) protein in complete Freund's adjuvant were injected by intraperitoneal (i.p.) route to 6- to 8-week-old BALB/c mice each for priming. Two weeks after the first injection, the mice were injected with another 25 *μ*g of rrHDGF protein in PBS. The procedures were repeated twice on week 4 and 5 after antigen priming. The sera were collected and stored at -20°C until use. The capability of rHDGF antibody from mice has a blocking effect that can inhibit the activity of HDGF.

### 2.3. Cell Culture

The PC-12 cells (BCRC 60048) were cultured in RPMI-1640 medium (Gibco) supplemented with 10% horse serum (Gibco) and 5% fetal bovine serum (Gibco) maintained in a humidified atmosphere containing 5% CO_2_ at 37°C.

### 2.4. MTT Assay

Treatment of PC12 cells for 14 days with 50 ng/ml nerve growth factor induction of the neuronal phenotype when plated on Collagen IV coated culture flask. These PC-12 cells (1 × 10^4^ cells/well in 24-well plates) were incubated with different concentrations of rHDGF antibody (0, 10, 20, 50, and 100 ng/ml) for 24 hours, and the medium was removed afterwards. Furthermore, the cells were treated with 500 *μ*M H_2_O_2_ for 24 hours. At the end of the treatment, 3-(4, 5-dimethylthiazol-2-yl)-2, 5-diphenyltetrazolium bromide (MTT) were added to each well at a final concentration of 0.5 mg/ml, and these cells were then incubated for another 90 minutes. The medium was then removed, and the purple formazan crystals were dissolved in DMSO (200 *μ*/well). Absorbance was determined with a Multiskan FC microplate photometer reader (Thermo Scientific) with a test wavelength of 570 nm and a reference wavelength of 620 nm to obtain the sample signal (OD570–OD620).

### 2.5. Animal Model

Thirty-six male Sprague-Dawley rats weighing 300-350 g were purchased from the National Animal Center (Taiwan). From the time of their arrival at the laboratory until completion of the experiment, the rats were housed in plastic cages with a temperature of 22 + 1°C, a relative humidity of 70%, a 12-hr light/12-hr dark cycle, and ad libitum access to normal food and water.

The rats were divided into three groups (*n* = 12/group). In the RTX group, neuropathy was induced by intraperitoneal administration of a single dose of RTX (50 *μ*g/kg, Sigma, St. Louis, MO) as described in the literature [9, 27, 28]. In the RTX+rHDGF antibody group, 100 *μ*g/kg rHDGF antibody was administered and applied 24 hours before RTX injection. The control group received no rHDGF antibody pretreatment and no RTX injection.

All procedures were performed according to ethical guidelines for the care and use of laboratory animals [29], and the protocol for use of the experimental animals in this study was approved by the Institutional Animal Care and Use Committee of Kaohsiung Medical University. All experimental procedures were carefully performed to minimize the suffering of the laboratory animals.

### 2.6. Calibrated Forceps Testing

Calibrated forceps (Rodent Pincher-Analgesia Meter; Bioseb In Vivo Research Instruments, Vitrolles, France) were used to apply mechanical stimulation in the experimental animals, and the mechanical threshold response was measured with an algometer and quantified on a linear scale. The maximum force (g) applied to the paw at the time of withdrawal was recorded with a dynamometer. For sensitive and reliable measurements, mechanical threshold was measured three times in each hindpaw as previously described by Luis-Delgado et al. [30]. All measurements were obtained by a single trained technician who was blinded to the experiment group.

### 2.7. Immunofluorescence Staining

Transversal frozen sections (8 *μ*m) of spinal cord (L3-L5) were dried and incubated in a blocking buffer containing 1.5% normal goat serum and 0.2% Triton X-100 in PBS. The sections were washed with PBS twice, incubated overnight with the primary antibodies [GFAP (1 : 1000; BD Pharmingen™; 55639), Neu-N (1 : 1000; Millipore; MAB377)] at 4°C, washed repeatedly with PBS, and finally replaced in secondary antibodies conjugated with Alexa 488 (1 : 1000; Millipore) or Cy3 (1 : 1000; Millipore) for 3 h at room temperature. Each image from 3 to 5 sections stained at least from 6 rats for each group was photographed under a confocal microscope. The total numbers of apoptotic neuron cells were obtained manually from 5 tissue sections. The mean intensity of GFAP were determined from 5-randomly selected fields of each tissue section (laminae I-II of spinal cord dorsal horn) through Image J.

### 2.8. Terminal Deoxynucleotidyl Transferase- (TdT-) Mediated dUTP Nick End Labeling (TUNEL) Assay

Cell death was assessed by TUNEL assay (Millipore; S7110). A mixture of 1 *μ*l TdT enzyme, 45 *μ*l equilibrium buffer, and 5 *μ*l nucleotide mix was prepared in the dark, added onto each slide, and incubated at 37°C for 1-2 hours in the dark. The enzymatic reaction was stopped by adding 4 *μ*l of SSC (2X) onto each slide and then incubating for another 15 minutes at room temperature. Unbound fluorescent-12-dUTP was removed by washing with PBS. The cells were stained by immersing the slides in propidium iodide for 15 minutes in the dark. The slides were dried after rinsing with deionized water and then overlaid with cover slips. The TUNEL positive cells were observed under a fluorescence microscope (Olympus, U-RFL-T).

### 2.9. Western Blot

For protein extraction, the spinal cord dorsal horn tissues (L3-L5) were homogenized in protein lysis buffer in the presence of protease inhibitors (Sigma) and incubated at room temperature for 30 minutes. Samples were centrifuged at 13,000 × rpm for 30 minutes at 4°C. Protein concentrations in the supernatants were determined with a Bio-Rad DC Protein Assay Kit. For Western blot analysis, an equal amount of total proteins was separated by 12% sodium dodecyl-sulfate polyacrylamide gel electrophoresis and transferred onto polyvinylidene fluoride membranes. After blocking in Tris-buffered saline containing 0.05% Tween-20 and 5% nonfat milk for 1 hour at room temperature, the membranes were incubated overnight at 4°C with various primary antibodies [iNOS (1 : 500; BD; 610328), TrkB (1 : 500; ABGENT; AN1211), PI3K (1 : 500; ABGENT; AP52796), Akt (1 : 500; Cell Signaling; #9272), p-Akt (1 : 500; Cell Signaling; #9271), substance P (1 : 500; Abcam; ab10353), HDGF (1 : 500; proteintech; 60064-1-lg), and *β*-actin (1 : 20000; Sigma; A5441)] directed against the protein of interest. After several washes, an appropriate HRP-conjugated secondary antibody (1 : 5000; Millipore; P36599A) was applied for 1 hour at room temperature. Peroxidase activity was visualized with an ECL Western Blotting Detection kit and a Mini Chemi professional machine (Sage Creation Science, Co., Ltd., Beijing, China).

### 2.10. Enzyme-Linked Immunosorbent Assay (ELISA)

High sensitivity ELISA was performed to assess HDGF levels in serum (Elabscience; E-EL-H5651). A 96-well plate was coated overnight in carbonate coating buffer, blocked in the provided sample buffer for 2 hours at room temperature, and treated with human HDGF antibody for 2 hours at room temperature. Acid-treated samples and provided standards were added to the plate in duplicate. Wells were then treated with anti-IgY conjugated to HRP for 1 hour at room temperature, and color was developed with provided tetrametylbenzidine dihydrochloride solution for 10 mins and stopped with 1 N HCl. The absorbance of wells was measured at 450 nm. The HDGF concentration was determined by comparing mean absorbance between duplicate samples and standards. The HDGF concentration was then normalized to total protein content and expressed as ng/*μ*l total protein.

### 2.11. Reverse Transcription-Polymerase Chain Reaction (RT-PCR)

Total RNA from DRG was isolated with an SV Total RNA Isolation System (Promega, Madison, WI). The integrity of the isolated RNA was confirmed by agarose gel electrophoresis. The RNA concentration was determined by spectrophotometry. For RT, 1-*μ*g samples of total RNA were reverse transcribed into cDNA in 20 *μ*l of reaction mixture containing 200 units of Superscript II (Gibco BRL, Rockville, MD), 0.5 *μ*g oligo(dT)12-18, 200 pmol dithiothreitol, 10 pmol dNTP, and 40 units of ribonuclease inhibitor (Gibco BRL, Life Technologies) in a buffer supplied with the enzymes. The RT procedure was performed according to the protocol suggested by the manufacturer (Superscript II; Gibco BRL). The resulting cDNA was frozen at -20°C for mRNA detection. Target genes were amplified by PCR performed with a Taq DNA polymerase kit (Fast Start Taq DNA polymerase; Roche Diagnostics GmbH, Mannheim, Germany). Next, 1.5 *μ*l cDNA from RT was replicated in PCR reactions in a total volume of 25 *μ*l containing Taq DNA polymerase (0.63 units), buffer supplied with enzymes, MgCl_2_ (50 pmol), dNTP (200 *μ*mol), and primers (20 pmol). The PCR conditions were 1 cycle of 95°C for 20 seconds followed by 40 cycles of 95°C for 3 seconds and 60°C for 30 seconds. After completion of PCR, a final melting curve was performed by denaturation at 95°C for 15 seconds and then recorded by cooling to 60°C and then heating slowing until 95°C for 15 seconds. All primers were manufactured by Fisher Scientific. Primer of GAPDH (glyceraldehydes-3-phosphate dehydrogenase): forward sequence: 5′-AGACAGCCGCATCTTCTTGT-3′ and reverse sequence: 5′-CTTGCCGTGGGTAGAGTCAT-3′. Primers of TNF-*α*: forward sequence: 5′-GCCCAGACCCTCACACTC-3′ and reverse sequence: 5′-CACTCCAGCTGCTCCTCT-3′. Primer of IL-1*β*: forward sequence: 5′-CACCTTCTTTTCCTTCATCTTTG-3′ and reverse sequence: 5 ′-GTCGTTGCTTGT CTCTCCTTGTA-3 ′. The RT-PCR was performed on a Step One Plus Real-Time PCR system using the Step One software V2.3 (Applied Biosystems).

### 2.12. Data Analyses

All data from this study were expressed as mean + standard error of the mean (SEM). Differences between multiple groups were analyzed by analysis of variance (ANOVA) with two-tailed probabilities; multiple comparisons were performed by Tukey post hoc test. A *P* value less than 0.05 was considered statistically significant. Representative results for a least three independent experiments were recorded. All of the above analyses were performed with the SPSS statistical software (V24.0) (SPSS, IBM, New York) and GraphPad Prism 6.0 (GraphPad Software., CA, USA).

## 3. Results

### 3.1. HDGF Protein Expression in Sciatica

**Figure 1:**
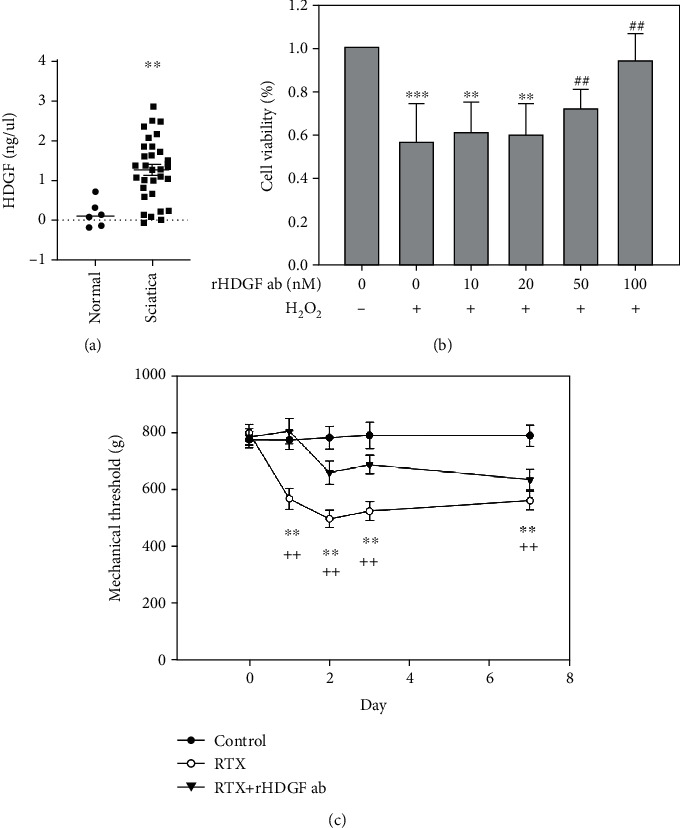
Serum HDGF is expressed higher in sciatica patients and rHDGF antibody increases pain threshold in RTX-treated rats. (a) Comparison of serum HDGF protein expression between sciatica patients and normal controls after ELISA analysis. The values detected from individuals were drawn as dots. The expression levels were shown as scattered plots; the middle line demonstrated the mean value. ^∗∗^*P* < 0.01 compared with normal controls. (b) The PC-12 cells were incubated with different concentrations of rHDGF antibody (0, 10, 20, 50, and 100 nM) and 500 *μ*M H_2_O_2_. Values are expressed as percentages of viable cells. ^∗∗^*P* < 0.01, ^∗∗∗^*P* < 0.001 compared with control group (without rHDGF antibody and H_2_O_2_). ^##^*P* < 0.01 compared with the control group treated with 500 *μ*M H_2_O_2_. Data are expressed as the mean + SEM (*n* = 6). (c) After the rats were divided into three groups, neuropathy was induced by intraperitoneally administration of a single dose of RTX (50 *μ*g/kg). In the RTX+rHDGF antibody group, 100 *μ*g/kg rHDGF antibody was administered and applied 24 hours before RTX injection. The control group received no rHDGF antibody pretreatment and no RTX injection. Calibrated forceps test was used to test the mechanical threshold for pain before and 1, 2, 3, and 7 days after RTX injection. ^∗∗^*P* < 0.01, compared with control group. ^++^*P* < 0.01 compared with RTX+rHDGF antibody group. Data are expressed as the mean + SEM (*n* = 6 per group).

To evaluate HDGF protein expression in patients with sciatica, serum samples were collected from 6 normal controls and from 31 patients with sciatica. The ELISA results showed that serum HDGF protein expression was significantly higher in the sciatica group (1.2725 + 0.79225 ng/*μ*l) compared to the control group (0.1664 + 0.32404 ng/*μ*l) (*P* < 0.01) (Figure 1(a)). This difference suggested that HDGF may have a crucial role in sciatica.

### 3.2. Blocking HDGF Had Protective Effects on Neuron Cells

To decide the concentration of rHDGF antibody with protective effect, we used PC-12 cells as neuron cells and MTT assay to detect the cell viability. In this study, we compared the different concentrations of 0, 10, 20, 50, and 100 nM rHDGF antibody with 500 *μ*M H_2_O_2_. The MTT assay results showed that the cell viability of control group treated with 500 *μ*M H_2_O_2_ was significantly lower that control group (without rHDGF antibody and H_2_O_2_) (*P* < 0.001). Treatment with 100 nM rHDGF antibody significantly protected the cell viability with 500 *μ*M H_2_O_2_ (*P* < 0.01) (Figure 1(b)). These data indicate that H_2_O_2_ decreased the viability of neuron cells whereas blocking HDGF protected neuron cell viability.

### 3.3. rHDGF Antibody Attenuated RTX-Induced Neuropathic Pain

The rat model was used to investigate how blocking HDGF affects neuropathic pain induced by intraperitoneally injection with RTX. Calibrated forceps test was performed to detect the mechanical threshold. The RTX group showed significant decreases in paw withdrawal thresholds from day 1 after RTX injection as compared with the control in the same group (*P* < 0.01). Moreover, the RTX+rHDGF antibody group showed significant increases in paw withdrawal thresholds (*P* < 0.01) as compared with RTX group (Figure 1(c)). That is, pretreatment with rHDGF antibody improved the mechanical threshold for neuropathic pain induced by RTX injection.

### 3.4. rHDGF Antibody Inhibited Astrocyte Reactions

**Figure 2:**
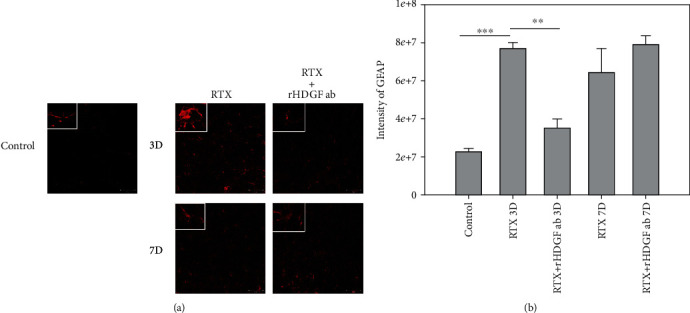
Effect of rHDGF antibody on astrocytes in the L3/L5 spinal cord dorsal horn (laminae I-II) of RTX-treated rats. After the rats were divided into three groups, neuropathy was induced by intraperitoneally administration of a single dose of RTX (50 *μ*g/kg). In the RTX+rHDGF antibody group, 100 *μ*g/kg rHDGF antibody was administered and applied 24 hours before RTX injection. The control group received no rHDGF antibody pretreatment and no RTX injection. At 3 or 7 days after RTX injection and/or pretreated with rHDGF antibody, L3-L5 spinal cord dorsal horn were harvested, and the proliferation of astrocytes (GFAP) was detected through immunofluorescence staining (200x magnification). (a) Representative immunofluorescence staining image shown are from 3 to 5 sections stained at least from 6 rats for each group. White rectangle in (a) indicates the astrocyte morphology for high magnifications. (b) Changes in intensity. ^∗∗^*P* < 0.01, ^∗∗∗^*P* < 0.001. Data are expressed as the mean + SEM (*n* = 6 per group).

Nerve injury from sciatic nerve ligation is known to activate glial reactions [31, 32]. Astrocyte reaction (GFAP upregulation and astrogliosis) has also been observed in various injury conditions associated with enhanced pain states [33]. Immunofluorescence staining for GFAP staining was used to detect reactive astrocytes. The staining results showed that RTX induced significant increases in the proliferation of astrocytes on day 3 and day 7 (*P* < 0.001). As well, the astrocytes became swollen after exposed to RTX (Figure 2(a)). Pretreatment with rHDGF antibody significantly inhibited proliferation of astrocytes as well as morphology on day 3 after RTX injection (*P* < 0.01) but not on day 7 after RTX injection (Figures 2(a) and 2(b)).

### 3.5. rHDGF Antibody Decreased Inflammatory Cytokines

**Figure 3:**
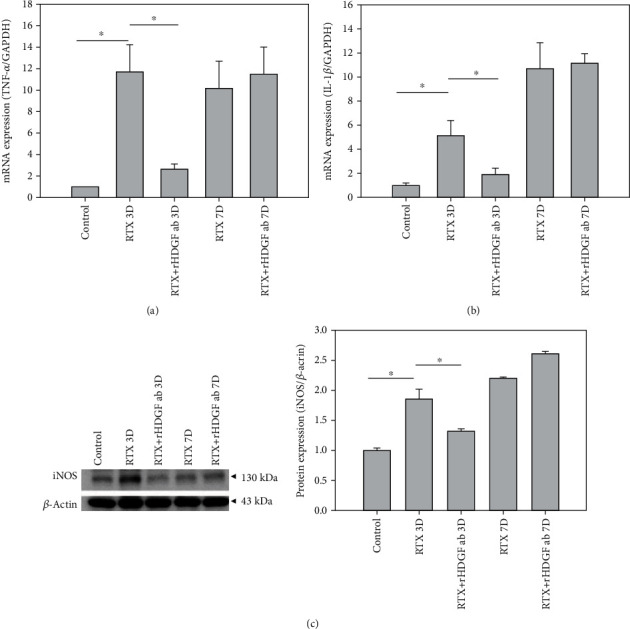
Effect of rHDGF antibody on inflammatory cytokines in RTX-treated rats. After the rats were divided into three groups, neuropathy was induced by intraperitoneally administration of a single dose of RTX (50 *μ*g/kg). In the RTX+rHDGF antibody group, 100 *μ*g/kg rHDGF antibody was administered and applied 24 hours before RTX injection. The control group received no rHDGF antibody pretreatment and no RTX injection. At 3 or 7 days after RTX injection and/or pretreated with rHDGF antibody, L3-L5 dorsal root ganglion and spinal cord dorsal horn were harvested. Inflammatory cytokines were examined through RT-PCR or western blot analysis. (a) Gene expression of TNF-*α* and (b) IL-1*β* in dorsal root ganglion of rats. Gene expressions are ratios relative to GAPDH. ^∗^*P* < 0.05. (c) iNOS expression in the spinal cord dorsal horn of rats. Representative Western blot results was shown. Expression levels were normalized to *β*-actin. ^∗^*P* < 0.05. Data are expressed as the mean + SEM (*n* = 6 per group).

Activated inflammatory mediators that develop after peripheral nerve injury may cause a series of cellular and molecular events [34, 35]. Inflammatory responses induced by RTX in DRG and the spinal cord dorsal horn were investigated by performing RT-PCR and western blot analysis. Compared to the control group, the RTX group had significantly higher mRNA expressions of TNF-*α* (Figure 3(a)) and IL-1*β* (Figure 3(b)) in DRG on day 3 (*P* < 0.05) and day 7 after RTX injection. In the RTX+rHDGF antibody group, pretreatment with rHDGF antibody significantly inhibited mRNA expression of TNF-*α* and IL-1*β* on day 3 (*P* < 0.05) after RTX injection but not on day 7 after RTX injection (Figures 3(a) and 3(b)). Western blot analysis of the spinal cord dorsal horn showed that protein expression of iNOS was significantly higher in the RTX group compared to the control group on day 3 after RTX injection (*P* < 0.05) and on day 7 after RTX injection (Figure 3(c)). Pretreatment with rHDGF antibody significantly inhibited the iNOS protein expression on day 3 (*P* < 0.05) but not on day 7 (Figure 3(c)).

### 3.6. rHDGF Antibody Inhibited Neuron Cells Apoptosis

**Figure 4:**
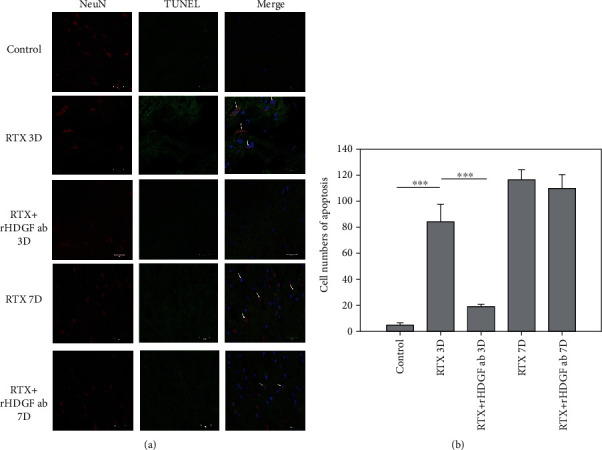
Effect of rHDGF antibody on neuron cells in the L3/L5 spinal cord dorsal horn of RTX-treated rats. After the rats were divided into three groups, neuropathy was induced by intraperitoneally administration of a single dose of RTX (50 *μ*g/kg). In the RTX+rHDGF antibody group, 100 *μ*g/kg rHDGF antibody was administered and applied 24 hours before RTX injection. The control group received no rHDGF antibody pretreatment and no RTX injection. At 3 or 7 days after RTX injection and/or pretreated with rHDGF antibody, L3-L5 spinal cord dorsal horn were harvested, and the expression of apoptotic neurons were evaluated through immunofluorescence staining by TUNEL assay (green), neuron cell marker (Neu-N) (Red), and DAPI (blue) (400x magnification). (a) Representative immunofluorescence staining image shown are from 3 to 5 sections stained at least from 6 rats for each group. Apoptotic neurons visualized (indicated by arrows) in the dorsal horn of the L3/L5 spinal cord are also shown. (b) The numbers of apoptotic neurons per animal were shown. ^∗∗∗^*P* < 0.001. Data are expressed as the mean + SEM (*n* = 6 per group).

Peripheral or central nerve injuries result in Wallerian degeneration of axons at a lesion site [35–37]. Double immunofluorescence staining for TUNEL assay and neuron cell marker (Neu-N) showed increased apoptotic neurons in the spinal cord dorsal horn in rats treated with RTX. In the spinal cord dorsal horn, RTX induced neuron cell apoptosis on day 3 (*P* < 0.001) and day 7 after RTX injection (Figure 4). Pretreatment with rHDGF antibody decreased the number of the apoptotic neuron cells in the spinal cord dorsal horn on day 3 (*P* < 0.001), but not on day 7 after RTX injection (Figure 4). These data indicate that blocking HDGF prevented neuron cell apoptosis induced by RTX in the spinal cord dorsal horn.

### 3.7. Effect of rHDGF Antibody on Protein Expressions Associated with Pain

**Figure 5:**
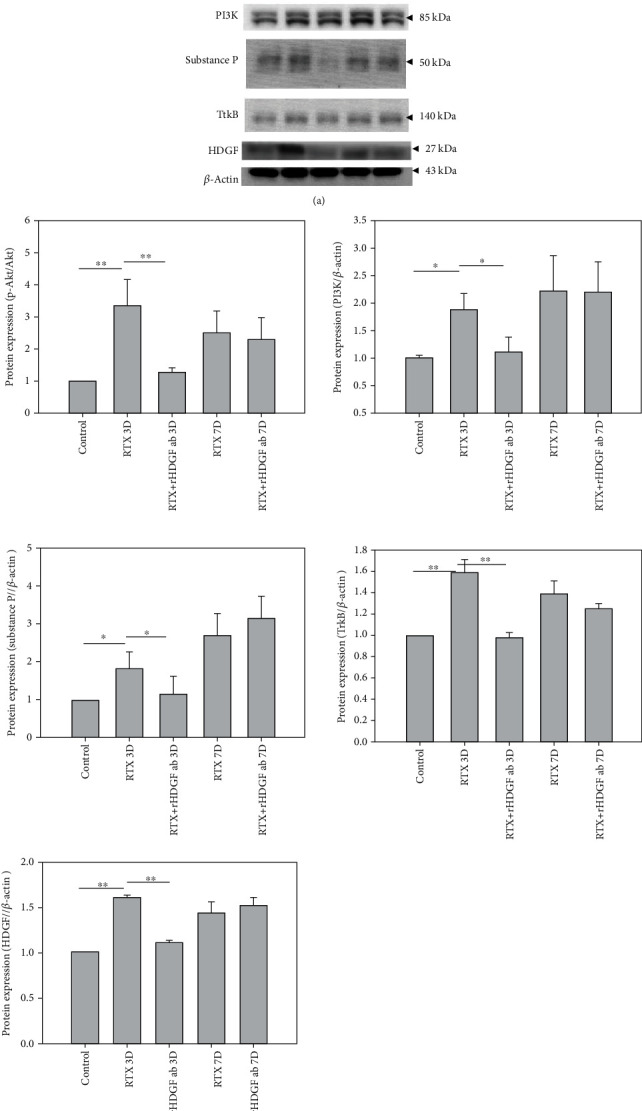
Effect of rHDGF antibody on expressions of HDGF, p-Akt/Akt, PI3K, substance P, and TrkB in the L3/L5 spinal cord dorsal horn of RTX-rats. After the rats were divided into three groups, neuropathy was induced by intraperitoneally administration of a single dose of RTX (50 *μ*g/kg). In the RTX+rHDGF antibody group, 100 *μ*g/kg rHDGF antibody was administered and applied 24 hours before RTX injection. The control group received no rHDGF antibody pretreatment and no RTX injection. At 3 or 7 days after RTX injection and/or pretreated with rHDGF antibody, L3-L5 spinal cord dorsal horn were harvested and protein expressions were measured through western blot analysis. (a) Representative Western blot results. (b) Expression levels were normalized to *β*-actin. ^∗^*P* < 0.05, ^∗∗^*P* < 0.01. Data are expressed as the mean + SEM (*n* = 6 per group).

The effects of blocking HDGF on RTX-induced neuropathic pain in the spinal cord dorsal horn were investigated by western blot analysis of expressions of proteins associated with pain, including HDGF, p-Akt/Akt, PI3K, substance P, and TrkB.  Figure 5 shows that, compared to the control group, the RTX group had significantly higher expressions of HDGF (*P* < 0.01), p-Akt/Akt (*P* < 0.01), PI3K (*P* < 0.05), substance P (*P* < 0.05), and TrkB (*P* < 0.01) on day 3 after RTX injection and significantly higher expressions of HDGF, p-Akt/Akt, PI3K, substance P, and TrkB on day 7 after RTX injection. In contrast, compared to the RTX group, the RTX+rHDGF antibody group had significantly lower expressions of HDGF (*P* < 0.01), p-Akt/Akt (*P* < 0.01), PI3K (*P* < 0.05), substance P (*P* < 0.05), and TrkB (*P* < 0.01) on day 3. On day 7 after RTX injection, however, the RTX+rHDGF antibody group did not reveal significantly lower expressions of HDGF, p-Akt/Akt, PI3K, substance P, or TrkB.

## 4. Discussion

The comparisons in this study revealed significantly higher levels of serum HDGF in the sciatica patients compared to normal controls. The rat model of neuropathy used in this study showed that RTX injections decreased the mechanical threshold for pain, upregulated TNF-*α* inflammation in the DRG, and upregulated expressions of HDGF, PI3K, p-Akt/Akt, TrkB, iNOS, and substance P in the spinal cord. The model obtained structural evidence of the role of central nervous system sensitization in the development of neuropathic pain after RTX injection. The HDGF is a growth factor and a mitogen with proliferative effect in various cells. The experiments demonstrated that blocking HDGF can reverse molecular events associated with the perception of pain induced by TNF-*α*-mediated inflammation.

Glial alteration is a unifying mechanism of persistent hypersensitivity in chronic pain [38–40]. Many models agree that changes in glial phenotype mainly occur in the spinal cord, higher brain regions [41], and the peripheral nervous system [12, 42]. In the spinal cord, glial cells are responsible for functional and structural modifications. Activation of astrocytes may be responsible for long term maintenance of chronic pain [43]. Glial cells produce and release the proinflammatory cytokines, which can directly act on their receptors expressed on superficial dorsal horn neurons through synaptic mechanisms such as neural-glial interaction. Therefore, they sensitize the nociceptive pathway by enhancing excitatory synaptic transmission and by reducing inhibitory synaptic transmission [44, 45]. Inflammatory responses in the peripheral and central nervous systems are known to play crucial roles in the development and persistence of pathological pain states [46]. The proinflammatory cytokine TNF-*α* is expressed in various cell types, including neuronal and immune cells. Following nerve injury, TNF-*α* is synthesized by glial cells in the central nervous system and by Schwann cells in the peripheral nervous system [12]. Additionally, TNF-*α* is expressed in DRG neurons and is upregulated after peripheral nerve injury [47, 48]. Many studies suggest that TNF-*α* initiates and modulates neuronal activity in various classes of neurons and peripheral axons [48, 49]. Furthermore, TNF-*α* modulates nociceptive stimuli by enhancing downstream N-Methyl-D-aspartate (NMDA) currents [44]. Intraganglional application of TNF-*α* induces thermal hyperalgesia and sustained mechanical allodynia in rats [50, 51]. The increased expression of TNF-*α* induced by pain stimuli in nervous tissue indicates its essential role in the sensation of pain. Production of TNF-*α* starts as soon as 1 hour after injury and can continue for up to 2 weeks before decreasing. Fluctuation in TNF-*α* levels tends to increase or decrease with states of mechanical allodynia and thermal hyperalgesia [52]. Our rat model revealed that RTX induced proliferation of astrocytes and inflammatory cytokines on day 3 and day 7 after injection, and the proliferating effects were consistent with changes in pain behavior. In the spinal cord dorsal horn, blocking HDGF inhibited the proliferation of glial cells and inhibited the release of inflammatory factors.

The important role of apoptosis in neuropathic pain is well established. Peripheral nerve injury causes neuronal apoptosis in the spinal cord dorsal horn [53, 54], which was consistent with our study shown on day 3 and day 7 after injection of RTX. The mechanical threshold of neuropathic pain significantly decreased with changes in apoptosis beginning on day 1 after RTX injection and continued until day 7. This suggests that neuron cell apoptosis has an important contributing role in persistent neuropathic pain. Binding between TNF-*α* and TNF receptors causes recruitment of TNFR1-associated protein and release of Smac/Diablo from mitochondria and is a hypothesized cause of neuronal cell death [52, 55]. Studies show that silencing HDGF prevents TNF-*α*-induced release of proapoptotic factors from mitochondria [11]. However, other studies have reported that an HDGF knockdown can induce apoptosis [56] and cell cycle arrest in several human cancers. A HDGF knockdown can also induce apoptosis through a Fas-mediated extrinsic apoptotic pathway and can induce apoptosis through a Bad-mediated intrinsic apoptotic pathway when ERK and Akt are inactivated [57, 58]. Our study showed that pretreatment with rHDGF antibody decreased the number of the apoptotic neuron cells in the spinal cord dorsal horn on day 3 after neuropathic pain was experimentally induced by injection of RTX.

Neuropathic pain induced by peripheral or central nerve injury evokes various changes in biological and biochemical markers [59–61]. Central and peripheral sensitization ensue after many inflammatory processes [62]. The PI3K signal transducer enzyme is involved in various physiological and pathological functions. It activates plasma membrane localized protein kinase (Akt) and is a key mediator of central sensitization of spinal cord neurons associated with persistent afferent inputs. This enzyme also contributes to chronic pain and has been implicated in NMDA receptors and wind-up of dorsal horn nociceptive neurons [63–65]. Production of NO is triggered by calcium influx into neurons after NMDA receptors are opened [64]. The PI3K/Akt signaling reportedly participates in activation of NOS [66–69]. Of all isoforms, iNOS has the most important roles in inflammation and pain. In high concentrations, iNOS is neurotoxic and can trigger Wallerian degeneration [70]. Damage to peripheral sensory neurons then affect the central nervous system through release of neurotransmitters, including glutamate, substance P, and brain-derived neuropathic factor (BDNF), from the central terminals in the primary nociceptor afferents [71–73]. Binding of BDNF to tyrosine kinase receptor in the post synaptic membrane in turn mediates phosphorylation of NMDA receptors, which is associated with onset and maintenance of neuropathic pain [74, 75]. Substance P coexists with glutamate and plays a crucial role in pain perception. Since a release of substance P induced by noxious simulation can modulate NMDA receptors, substance P promotes central hyperexcitability and increases pain sensitivity [62, 72, 76]. Our study showed that expressions of proteins PI3K, p-Akt/Akt, TrkB, iNOS, and substance P were significantly higher in the RTX group compared to the control group. Pretreatment with rHDGF antibody significantly decreased expressions of proteins PI3K, p-Akt/Akt, TrkB, iNOS, and substance P on day 3 after RTX injection.

## 5. Conclusions

Our results suggest that rHDGF antibody protects against RTX-induced neuropathic pain mediating inflammation induced by proinflammatory cytokines. The serum level of HDGF was higher in sciatica patients compared to normal controls. Our animal model of RTX-induced neuropathic pain revealed that higher protein expression of HDGF in spinal cord and blocking HDGF improved the mechanical threshold response. In DRG, blocking HDGF inhibited inflammatory cytokines. In the spinal cord dorsal horn, blocking HDGF inhibited proliferation of astrocytes and inhibited apoptosis of neuronal cells. Additionally, blocking HDGF attenuated expressions of proteins associated with pain (iNOS, TrkB, substance P, PI3K, and p-Akt/Akt). Taken together, the experimental results indicate that inhibiting HDGF can reduce neuropathic pain caused by an inflammatory response.

## Data Availability

The data used to support the findings of this study are included within the article.
